# Supplementary material for: Factors influencing drug-susceptible tuberculosis treatment outcomes in Romania and Ukraine
Source: PLoS One. 2025 Dec 3;20(12):e0337937. doi: 10.1371/journal.pone.0337937 (PMC12674542; doi:10.1371/journal.pone.0337937)
Supplement: S1 Box — (PDF) [file pone.0337937.s001.pdf]

Box S1. Outcome definitions

New case - have never been treated for TB or have taken anti-TB drugs for less than 1 month

Retreatment - a patient who has undergone TB treatment in the past. Rapid relapse - a patient who has been treated for TB under 2 years ago

Slow response - either smear positive pulmonary TB patients with sputum smear not decreasing adequately (4+ to 2+, 3+ to 1+, 2+/1+ to negativity) or not negative at the end of the intensive phase or no clinical improvement (no weight gain, no reduction in cough, persistent fever, worsening of chest X-Ray)

Treatment success - cured, treatment completed. Cured - a pulmonary TB patient with bacteriologically confirmed TB at the beginning of treatment who was smear- or culture-negative in the last month of treatment and on at least one previous occasion. Treatment completed - TB patient who completed treatment without evidence of failure BUT with no record to show that sputum smear or culture results in the last month of treatment and on at least one previous occasion were negative, either because tests were not done or because results are unavailable.

Unfavourable outcomes:

- Treatment failed - TB patient whose sputum smear or culture is positive at month 5 or later during treatment.
- Died - TB patient who dies for any reason before starting or during the course of treatment.
- Lost to follow-up - TB patient who did not start treatment or whose treatment was interrupted for 2 consecutive months or more.

Not evaluated - TB patient for whom no treatment outcome is assigned. This includes cases “transferred out” to another treatment unit as well as cases for whom the treatment outcome is unknown to the reporting unit.
