# Supplementary material for: Factors influencing drug-susceptible tuberculosis treatment outcomes in Romania and Ukraine
Source: PLoS One. 2025 Dec 3;20(12):e0337937. doi: 10.1371/journal.pone.0337937 (PMC12674542; doi:10.1371/journal.pone.0337937)
Supplement: S1 Table — (PDF) [file pone.0337937.s002.pdf]

Table S1. Univariate logistic regression analyses for unfavourable outcomes

Characteristics	Unfavourable outcome N=101	Favourable outcome N=737	Unadjusted OR (95% CI)	p-value
**Age**				
Under 35	14 (13.9%)	187 (25.4%)	1	-
35-under 65	55 (54.5%)	418 (56.7%)	1.75 (.95-3.24)	0.07
Over 65	32 (31.7%)	132 (17.9%)	3.23 (1.66-6.30)	0.001
**Gender**				
Male	72 (71.3%)	485 (65.8%)	1.29 (.81-2.03)	0.28
Female	29 (28.7%)	252 (34.2%)	1	-
**Living situation**				
Urban	36 (35.6%)	304 (41.3%)	1	-
Rural	59 (58.4%)	427 (58%)	1.16 (0.75-1.81)	0.49
Homeless	6 (5.9%)	5 (0.7%)	10.13 (2.94-34.88)	0.000
**Days hospitalisation**				
<=30 days	50 (49.5%)	230 (31.2%)	1	-
>30 days	51 (50.5%)	507 (68.8%)	0.46 (.30-.70)	<0.001
**Location of TB**				
Pulmonary	76 (75.2%)	530 (71.9%)	1	-
Extrapulmonary	16 (15.8%)	123 (16.7%)	0.91 (.51-1.61)	0.74
Combination	9 (8.9%)	84 (11.4%)	0.75 (.36-1.55)	0.43
**Previous TB**				
New case	73 (72.3%)	636 (86.3%)	1	-
Prev TB over 2 years ago	17 (16.8%)	82 (11.1%)	1.80 (1.01-3.21)	0.04
Prev TB under 2 years ago	11 (10.9%)	19 (2.6%)	5.04 (2.31-11.01)	<0.001
**Smoking**				
Never	28 (30.8%)	255 (38.1%)		
Former	9 (9.9%)	69 (10.3%)	1.18 (0.54-2.64)	0.67
Current	54 (59.3%)	346 (51.6%)	1.42 (0.88-2.30)	0.15
**Alcohol**				
Never	25 (28.7%)	244 (43%)	1	-
Light drinking	16 (18.4%)	116 (20.4%)	1.35 (0.69-2.62)	0.38
Moderate drinking	13 (14.9%)	86 (15.1%)	1.48 (0.72-3.01)	0.28
Heavy drinking	33 (37.9%)	122 (21.5%)	2.64 (1.50-4.63)	0.001
**Number of chronic conditions**				
None	90 (89.1%)	702 (95.3%)	1	-
1-2	11 (10.9%)	35 (4.7%)	2.45 (1.20-4.99)	0.01
>= 3	0	0	-	-
**PLHIV**				
No	98 (97%)	721 (97.8%)		

Characteristics	Unfavourable outcome N=101	Favourable outcome N=737	Unadjusted OR (95% CI)	p-value
Yes	3 (3%)	16 (2.2%)	0.73 (0.21-2.53)	0.61
**Cirrhosis**				
No	95 (94.1%)	720 (97.7%)	1	-
Yes	6 (5.9%)	17 (2.3%)	0.37 (0.14-.97)	0.61
**Diabetes mellitus**				
No	98 (97%)	685 (92.9%)	1	-
Yes	3 (3%)	52 (7.1%)	2.48 (0.76-8.09)	0.13
**COPD**				
No	85 (84.2%)	647 (87.8%)	1	-
Yes	16 (15.8%)	90 (12.2%)	.74 (.42-1.31)	.30
**Asthma**				
No	98 (97%)	728 (98.8%)	1	-
Yes	3 (3%)	9 (1.2%)	0.40 (0.11-1.52)	0.18
**Cancer**				
No	98 (97%)	709 (96.2%)	1	-
Yes	3 (3%)	28 (3.8%)	1.29 (0.39-4.32)	0.68
**Cardio-vascular**				
No	66 (65.3%)	549 (74.5%)	1	-
Yes	35 (34.7%)	188 (25.5%)	0.65 (0.42-1.01)	0.05
**Gastroenterological**				
No	86 (85.1%)	649 (88.1%)	1	-
Yes	15 (14.9%)	88 (11.9%)	0.78 (.43-1.41)	0.40
**Chronic Kidney Disease**				
No	93 (92.1%)	727 (98.6%)	1	-
Yes	8 (7.9%)	10 (1.4%)	0.16 (.06-.42)	<0.001
**BMI**				
Normal weight	49 (48.5%)	532 (72.2%)	1	-
Underweight	38 (37.6%)	122 (16.6%)	3.38 (2.12-5.39)	<0.001
Overweight	8 (7.9%)	64 (8.7%)	1.36 (0.62-2.99)	0.45
Obese	6 (5.9%)	19 (2.6%)	3.42 (1.31-8.98)	0.01
**TGO/ALAT Start (U/L)**				
Normal (under 40 U/L)	74 (73.3%)	626 (84.9%)	1	-
<3x normal	20 (19.8%)	99 (13.4%)	1.71 (0.99-2.92)	0.05
3x-10x normal	7 (6.9%)	11 (1.5%)	5.38 (2.02-14.31)	0.001
>10x normal	0	1 (0.1%)	-	-
**TGP/ASAT Start (U/L)**				
Normal (under 56 U/L)	88 (87.1%)	679 (92.1%)	1	-
<3x normal	11 (10.9%)	52 (7.1%)	1.63 (0.82-3.25)	0.16
3x-10x normal	2 (2%)	6 (0.8%)	2.57 (0.51-12.94)	0.25

Characteristics	Unfavourable outcome N=101	Favourable outcome N=737	Unadjusted OR (95% CI)	p-value
>10x normal	0	0	-	-
**TGO/ALAT Max Hosp (U/L)**				
Normal (under 40 U/L)	63 (62.4%)	539 (73.2%)	1	-
<3x normal	25 (24.8%)	149 (20.2%)	1.44 (0.87-2.36)	0.15
3x-10x normal	9 (8.9%)	39 (5.3%)	1.97 (0.91-2.36)	0.08
>10x normal	4 (4%)	9 (1.2%)	3.80 (1.13-12.71)	0.03
**TGP/ASAT Max Hosp (U/L)**				
Normal (under 56 U/L)	83 (82.8%)	610 (82.8%)	1	-
<3x normal	12 (12%)	94 (12.8%)	0.94 (0.49-1.79)	0.85
3x-10x normal	4 (4%)	29 (3.9%)	1.01 (0.35-2.96)	0.98
>10x normal	1 (1%)	4 (0.5%)	1.84 (0.20-16.64)	0.59
**eGFR Start (ml/min/1.73m2, CKD-EPI)**				
Normal (over 60)	89 (88.1%)	692 (93.9%)	1	-
60-15	9 (8.9%)	43 (5.8%)	1.63 (0.77-3.45)	0.21
Under 15	3 (3%)	2 (0.3%)	11.66 (1.92-20.75)	0.008
**eGFR Min Hosp (ml/min/1.73m2, CKD-EPI)**				
Normal (over 60)	84 (83.2%)	685 (93.3%)	1	-
60-15	12 (11.9%)	45 (6.1%)	2.18 (1.11-4.28)	0.02
Under 15	5 (5%)	4 (.5%)	10.19 (2.69-38.70)	0.001
