# Supplementary material for: Factors influencing drug-susceptible tuberculosis treatment outcomes in Romania and Ukraine
Source: PLoS One. 2025 Dec 3;20(12):e0337937. doi: 10.1371/journal.pone.0337937 (PMC12674542; doi:10.1371/journal.pone.0337937)
Supplement: S2 Table — (PDF) [file pone.0337937.s003.pdf]

Table S2: Univariate logistic regression analyses for loss to follow-up (LTFU)

Characteristics	LTFU N=42	Non-LTFU N=741	Unadjusted OR (95% CI)	p-value
**Age**				
Under 35	11 (26.2%)	188 (25.4%)	1	-
35-under 65	24 (57.1%)	421 (56.8%)	0.97 (0.46-2.03)	0.94
Over 65	7 (16.7%)	132 (17.8%)	0.90 (0.34-2.39)	0.90
**Gender**				
Male	28 (66.7%)	489 (66%)	1.03 (0.53-1.99)	0.92
Female	14 (33.3%)	252 (34%)	1	-
**Living situation**				
Urban	15 (35.7%)	306 (41.4%)	1	-
Rural	24 (57.1%)	429 (58%)	1.14 (0.59-2.21)	0.69
Homeless	3 (7%)	5 (0.7%)	12.24 (2.67-56.09)	0.001
**Days hospitalisation**				
<=30 days	15 (35.7%)	230 (31%)	1	-
>30 days	27 (64.3%)	511 (69%)	0.81 (0.42-1.55)	0.52
**Location of TB**				
Pulmonary	31 (73.8%)	533 (71.9%)	1	-
Extrapulmonary	9 (21.4%)	123 (16.6%)	1.26 (0.58-2.71)	0.55
Combination	2 (4.8%)	85 (11.5%)	0.41 (0.09-1.72)	0.22
**Previous TB**				
New case	29 (69%)	639 (86.2%)	1	
Prev TB over 2 years ago	7 (16.7%)	83 (11.2%)	1.86 (0.78-4.38)	0.16
Prev TB under 2 years ago	6 (14.3%)	19 (2.6%)	6.95 (2.59-18.73)	0.001
**Smoking**				
Never	13 (34.2%)	256 (38%)	1	
Former	2 (5.3%)	69 (10.2%)	0.57 (0.13-2.59)	0.47
Current	23 (60.5%)	349 (51.8%)	1.30 (0.64-2.61)	0.47
**Alcohol**				
Never	8 (22.9%)	244 (42.7%)	1	
Light drinking	10 (28.6%)	116 (20.3%)	2.62 (1.01-6.83)	0.047
Moderate drinking	7 (20%)	87 (15.2%)	2.45 (0.86-6.96)	0.092
Heavy drinking	10 (28.6%)	124 (21.7%)	2.46 (0.94-6.39)	0.065
**Number of chronic conditions**				
None	41 (97.6%)	706 (95.3%)	1	-
1-2	1 (2.4%)	35 (4.7%)	0.49 (0.67-3.68)	0.49
>= 3	0	0	-	-
**PLHIV**				
No	41 (97.6%)	725 (97.8%)	1	-
Yes	1 (2.4%)	16 (2.2%)	0.90 (0.12-6.99)	0.92

Characteristics	LTFU<br>N=42	Non-LTFU<br>N=741	Unadjusted OR<br>(95% CI)	p-value
**Cirrhosis**				
No	41 (97.6%)	724 (97.7%)	1	
Yes	1 (2.4%)	17 (2.3%)	0.96 (0.12-7.41)	0.97
**Diabetes mellitus**				
No	42 (100%)	689 (93%)	1	
Yes	0	52 (7%)	-	
**COPD**				
No	32 (76.2%)	651 (87.9%)	1	
Yes	10 (23.8%)	90 (12.1%)	0.44 (0.21-.93)	0.032
**Asthma**				
No	40 (95.2%)	732 (98.8%)	1	-
Yes		9 (1.2%)	0.25 (0.05-1.17)	0.079
**Cancer**				
No	42 (100%)	713 (96.2%)	1	-
Yes	0	28 (3.8%)	NA	NA
**Cardio-vascular**				
No	29 (69%)	662 (74.5%)	1	
Yes	13 (31%)	189 (25.5%)	0.76 (0.39-1.50)	0.43
**Gastroenterological**				
No	39 (92.9%)	653 (88.1%)	1	-
Yes	3 (7.1%)	88 (11.9%)	1.75 (0.53-5.79)	0.35
**Chronic Kidney Disease**				
No	40 (95.2%)	731 (98.7%)	1	-
Yes	2 (4.8%)	10 (1.3%)	0.27 (0.06-1.29)	0.10
**BMI**				
Normal weight	27 (64.3%)	535 (72.2%)	1	-
Underweight	8 (19%)	123 (16.6%)	1.29 (0.57-2.90)	0.54
Overweight	2 (4.8%)	64 (8.6%)	0.61 (0.14-2.67)	0.52
Obese	5 (11.9%)	19 (2.6%)	5.21 (1.81-15.02)	0.002
**TGO/ALAT Start (U/L)**				
Normal (under 40 U/L)	35 (83.3%)	628 (84.8%)	1	-
<3x normal	5 (11.9%)	101 (13.6%)	0.88 (0.34-2.32)	0.80
3x-10x normal	2 (4.8%)	11 (1.5%)	3.26 (0.69-15.29)	0.13
>10x normal	0	1 (0.1%)	-	
**TGP/ASAT Start (U/L)**				
Normal (under 56 U/L)	38 (90.5%)	683 (92.2%)	1	-
<3x normal	3 (7.1%)	52 (7%)	1.03 (0.31-3.47)	0.95
3x-10x normal	1 (2.4%)	6 (0.8%)	2.99 (0.35-25.51)	0.31
>10x normal	0	0	-	-

Characteristics	LTFU<br>N=42	Non-LTFU<br>N=741	Unadjusted OR<br>(95% CI)	p-value
**TGO/ALAT Max Hosp (U/L)**				
Normal (under 40 U/L)	31 (73.8%)	541 (73.1%)	1	
<3x normal	7 (16.7%)	151 (20.4%)	0.80 (0.34-1.87)	0.62
3x-10x normal	2 (4.8%)	39 (5.3%)	0.89 (0.20-3.87)	0.88
>10x normal	2 (4.8%)	9 (1.2%)	3.88 (0.80-18.72)	0.09
**TGP/ASAT Max Hosp (U/L)**				
Normal (under 56 U/L)	36 (87.8%)	614 (82.9%)	1	-
<3x normal	2 (4.9%)	94 (12.7%)	0.36 (0.08-1.53)	0.17
3x-10x normal	3 (7.3%)	29 (3.9%)	1.76 (0.51-6.06)	0.37
>10x normal	0	4 (0.5%)	-	-
**eGFR Start (ml/min/1.73m2, CKD-EPI)**				
Normal (over 60)	41 (87.6%)	696 (93.9%)	1	-
60-15	1 (2.4%)	43 (5.8%)	0.39 (0.05-2.93)	0.36
Under 15	0	2 (0.3%)	-	-
**eGFR Min Hosp (ml/min/1.73m2, CKD-EPI)**				
Normal (over 60)	39 (92.9%)	689 (93.4%)	1	-
60-15	3 (7.1%)	45 (6.1%)	1.18 (0.35-3.95)	0.79
Under 15	0	4 (0.5%)	-	-
