# Supplementary material for: Factors influencing drug-susceptible tuberculosis treatment outcomes in Romania and Ukraine
Source: PLoS One. 2025 Dec 3;20(12):e0337937. doi: 10.1371/journal.pone.0337937 (PMC12674542; doi:10.1371/journal.pone.0337937)
Supplement: S3 Table — (PDF) [file pone.0337937.s004.pdf]

Table S3. Univariate Cox regression analyses for death

Characteristics	Death<br>N=46	No death<br>N=756	Unadjusted HR<br>(95% CI)	p-value
**Age**				
Under 35	1 (2.2%)	190 (25.1%)	1	-
35-under 65	23 (50%)	429 (56.7%)	9.94 (1.34-73.59)	0.025
Over 65	22 (47.8%)	137 (18.1%)	27.96 (3.77-207.43)	0.001
**Gender**				
Male	34 (73.9%)	500 (66.1%)	1.43 (0.74-2.77)	0.29
Female	12 (26.1%)	256 (33.9%)	1	-
**Living situation**				
Urban	18 (39.1%)	311 (41.2%)	1	-
Rural	26 (56.5%)	438 (58%)	1.02 (0.56-1.86)	0.95
Homeless	2 (4.3%)	6 (0.8%)	4.99	0.03
**Days hospitalisation**				
<=30 days	32 (69.6%)	234 (31%)	1	-
>30 days	14 (30.4%)	522 (69%)	0.21 (0.11-0.39)	<0.001
**Location of TB**				
Pulmonary	34 (73.9%)	546 (72.2%)	1	-
Extrapulmonary	6 (13%)	124 (16.4%)	0.78 (0.33-1.87)	0.78
Combination	6 (13%)	86 (11.4%)	1.11 (0.47-2.65)	0.80
**Previous TB**				
New case	35 (76.1%)	648 (85.7%)	1	-
Prev TB over 2 years ago	8 (17.4%)	85 (11.2%)	1.70 (0.79-3.67)	0.17
Prev TB under 2 years ago	3 (6.5%)	23 (3%)	2.29 (0.70-7.44)	0.17
**Smoking**				
Never	12 (30%)	261 (37.9%)	1	-
Former	5 (12.5%)	71 (10.3%)	1.50 (0.53-4.27)	0.44
Current	23 (57.5%)	356 (51.7%)	1.39 (0.69-2.79)	0.36
**Alcohol**				
Never	14 (34.1%)	250 (42.7%)	1	-
Light drinking	4 (9.8%)	118 (20.1%)	0.61 (0.20-1.85)	0.38
Moderate drinking	4 (9.8%)	90 (15.4%)	0.80 (0.26-2.43)	0.80
Heavy drinking	19 (46.3%)	128 (21.8%)	2.54 (1.28-5.07)	0.008
**Number of chronic conditions**				
None	37 (80.4%)	720 (95.2%)	1	-
1-2	9 (19.6%)	36 (4.8%)	4.48 (2.16-9.29)	<0.001
>= 3	0	0	-	-
**PLHIV**				
No	45 (97.8%)	739 (97.8%)	1	-
Yes	1 (2.2%)	17 (2.2%)	1.03 (0.14-7.49)	0.98

Characteristics	Death<br>N=46	No death<br>N=756	Unadjusted HR<br>(95% CI)	p-value
**Cirrhosis**				
No	41 (89.1%)	738 (97.6%)	1	-
Yes	5 (10,9%)	18 (2.4%)	0.22 (0.09-.55)	0.001
**Diabetes mellitus**				
No	43 (93.5%)	704 (93.1%)	1	-
Yes	3 (6.5%)	52 (6.9%)	1.05 (0.33-3.38)	0.94
**COPD**				
No	85 (84.2%)	647 (87.8%)	1	-
Yes	16 (15.8%)	90 (12.2%)	1.15 (.46-2.92)	0.76
**Asthma**				
No	45 (97.8%)	745 (98.5%)	1	-
Yes	1 (2.2%)	11 (1.5%)	0.68 (0.09-4.94)	0.70
**Cancer**				
No	44 (95.7%)	727 (96.2%)	1	-
Yes	2 (4.3%)	29 (3.8%)	0.89 (0.22-3.66)	0.87
**Cardio-vascular**				
No	27 (58.7%)	563 (74.5%)	1	-
Yes	19 (41.3%)	193 (25.5%)	0.49 (0.28-.89)	0.02
**Gastroenterological**				
No	36 (78.3%)	666 (88.1%)	1	-
Yes	10 (21.7%)	90 (11.9%)	0.49 (0.25-1.00)	0.05
**Chronic Kidney Disease**				
No	40 (87%)	746 (98.7%)	1	-
Yes	6 (13%)	10 (1.3%)	0.12 (0.05-.28)	<0.001
**BMI**				
Normal weight	15 (32.6%)	540 (71.4%)	1	-
Underweight	26 (56.5%)	128 (16.9%)	6.69 (3.54-12.63)	<0.001
Overweight	4 (8.7%)	67 (8.9%)	2.11 (0.70-6.37)	0.18
Obese	1 (2.2%)	21 (2.8%)	1.72 (0.23-13.04)	0.59
**TGO/ALAT Start (U/L)**				
Normal (under 40 U/L)	32 (69.6%)	638 (84.4%)	1	-
<3x normal	9 (19.6%)	106 (14%)	1.67 (0.79-3.49)	0.18
3x-10x normal	5 (10.9%)	11 (1.5%)	7.73 (3.01-19.86)	<0.001
>10x normal	0	1 (1.5%)	-	-
**TGP/ASAT Start (U/L)**				
Normal (under 56 U/L)	41 (89.1%)	694 (91.8%)	1	-
<3x normal	4 (8.7%)	56 (7.4%)	1.20 (0.43-3.36)	0.72
3x-10x normal	1 (2.2%)	6 (0.8%)	2.67 (0.37-19.37)	0.33
>10x normal	0	0	-	-

Characteristics	Death<br>N=46	No death<br>N=756	Unadjusted HR<br>(95% CI)	p-value
**TGO/ALAT Max Hosp (U/L)**				
Normal (under 40 U/L)	27 (58.7%)	550 (72.8%)	1	-
<3x normal	11 (23.9%)	155 (20.5%)	1.42 (0.70-2.87)	0.32
3x-10x normal	7 (15.2%)	39 (5.2%)	3.47 (1.51-7.98)	0.003
>10x normal	1 (2.2%)	11 (1.5%)	1.85 (2.53-13.68)	0.54
**TGP/ASAT Max Hosp (U/L)**				
Normal (under 56 U/L)	38 (82.6%)	624 (82.6%)	1	-
<3x normal	7 (15.2%)	97 (12.8%)	1.18 (0.53-2.63)	0.69
3x-10x normal	1 (2.2%)	29 (2.8%)	0.58 (0.08-4.19)	0.58
>10x normal	0	5 (0.7%)	-	-
**eGFR Start (ml/min/1.73m2, CKD-EPI)**				
Normal (over 60)	36 (78.3%)	710 (93.9%)	1	-
60-15	7 (15.2%)	44 (5.8%)	3.04 (1.35-6.82)	0.007
Under 15	3 (6.5%)	2 (0.3%)	14.68 (4.51-47.76)	<0.001
**eGFR Min Hosp (ml/min/1.73m2, CKD-EPI)**				
Normal (over 60)	33 (71.7%)	703 (93.4%)	1	-
60-15	8 (17.4%)	46 (6.1%)	3.57 (1.65-7.73)	0.001
Under 15	5 (10.9%)	4 (0.5%)	15.07 (5.87-38.73)	<0.001
