# Supplementary material for: Factors influencing drug-susceptible tuberculosis treatment outcomes in Romania and Ukraine
Source: PLoS One. 2025 Dec 3;20(12):e0337937. doi: 10.1371/journal.pone.0337937 (PMC12674542; doi:10.1371/journal.pone.0337937)
Supplement: Attachment — Submitted filename: Reviewer Comments.docx [file pone.0337937.s006.docx]

**Reviewer Comments**

**Overall Assessment:**
This manuscript presents a valuable and well-conducted retrospective multi-center study analyzing predictors of unfavorable treatment outcomes in drug-susceptible tuberculosis (DS-TB) patients in two high-burden countries within the WHO European region: Romania and Ukraine. The topic is highly relevant, as identifying local predictors is crucial for tailoring interventions and improving TB control programs. The study is methodologically sound, with a clear design, robust statistical analysis, and a large, well-characterized cohort. The findings are novel for this specific regional context and have practical implications for clinicians and policymakers. I recommend acceptance after minor revisions to address the points outlined below.

**Abstract:**

1. Authors use the 2020 global TB mortality data in the abstract but 2022 data in the introduction. It is recommended that authors be consistent, preferably using the most recent (2022) data in both sections for accuracy.

**Introduction:**

1. The introduction establishes the global and regional context but would be strengthened by a more specific rationale for focusing on Romania and Ukraine. Please add a sentence stating the specific LTFU and death rates for DS-TB in these two countries (citing national reports or WHO data) to quantitatively establish the magnitude of the problem and the rationale for this study.

**Methods:**

1. Line 99: "follow up" should be "follow-up".
2. Please provide the rationale for selecting two centers in Romania and one in Ukraine. Furthermore, please state the proportion of patients contributed by each center in the Methods section (this data is in the Results, line 153-154) and discuss if any potential selection bias could arise from this uneven distribution.
3. Please clarify the sampling methodology. The manuscript states that patients "initiated treatment between 1 January 2019 to 31 December 2019." Does this imply that all consecutive eligible patients diagnosed within this one-year period were included? If not, what specific sampling or exclusion criteria were applied?
4. Lines 109 – 112: Kindly cite the references for the population statistics quoted for the study sites.
5. Lines 112 – 114: The reference for the statement that the region where Iasi is located has the lowest income in Romania should be cited.
6. Line 115: "Data collection" header is misaligned.
7. What data collection tool was used; was it adopted from the literature or was it self-developed by the authors? If it was self-developed, was it pre-tested or validated for this study?
8. Line 118: Authors mentioned that they collected “hospitalization data”. They should kindly explain what specific data this category encompassed and how it differed from the other data types mentioned (e.g., socio-demographics, lab parameters).
9. Lines 125-126: For clarity, please rephrase the data verification process. For example: "Data extraction was performed by a local investigator at each site. Subsequently, a second local investigator cross-verified the extracted data for accuracy and completeness."
10. Line 126: The initials of the team member who cross-checked the data (IM) should be deleted to maintain anonymity.
11. Lines 141 – 142: Inconsistent use of the symbol p and P. Authors should stick to the standard symbol of lowercase 'p' throughout the text.
12. Line 133 – 134: What is the reference for the age cut-off points used?

**Results:**

1. Line 158-159: "extrapulmonary TB cases involved the pleura (75.8%), either alone or in combination with pulmonary TB" – This is slightly confusing. It would be clearer to state the proportion of *all patients* who had pleural involvement.
2. Lines 183 – 184: How did authors ascertain the causality of adverse drug reactions to anti-TB drugs? What criteria (e.g., WHO-UMC, Naranjo) were used in determining causality and severity? Please provide the authors’ specific definition of “abnormal liver enzymes and renal function” and “persistently abnormal lab parameters” (e.g., values outside normal range for a specific duration).
3. Table 2: The header "Percentage, or median (IQR)*" is used, but the "Laboratory values" section lists counts and percentages, not medians. The table formatting could be adjusted for clarity.
4. The results for the multivariable models are presented clearly. However, for variables like "Age >65 years" and "Cavity present" in Table 3, which have p-values > 0.05 (0.157 and 0.196), it is important to reiterate in the text that they were retained due to the pre-specified model-building algorithm (P<0.2), even though they are not "statistically significant" at the conventional 0.05 level. Their effect sizes (aORs) are still notable.
5. Line 210: "hystory" should be "history".

**Discussion:**

1. Lines 232-234: The authors state the high success rate was achieved after hospitalizing all patients for a median of 39 days. This suggests a different standard of care. Please clarify in the Methods or Discussion: a) Is this hospitalization a mandatory national policy in Romania and Ukraine? b) What is the rationale for this approach? c) Does the median 39 days represent the entire treatment or just the initial intensive phase?
2. The discussion is somewhat fragmented and would benefit from a more structured comparison with existing literature. For each key finding (e.g., success rate, predictors like obesity/LTFU, culture conversion/LTFU), please first provide a direct comparison with data from similar studies, then offer plausible reasons for any agreement or divergence. The connection to similar studies (e.g., the reference to Russia) could be expanded.
3. Line 244 – 245: The statement that hospital-acquired infections such as *C. difficile* "depict drug toxicity" is inaccurate. *C. difficile* is a complication of antibiotic use due to microbiome disruption, not a direct toxic effect. This should be rephrased. Furthermore, please describe the method used to confirm causality of all adverse drug reactions and provide the data on the number and type of ADRs identified, including in which patient demographics they occurred.
4. **Line 248:** "clinical attitude" – consider rephrasing to "clinical management" or "clinical response".
5. Lines 248 – 251: The passage is confusing and risks conflating a process with an outcome. Please rephrase to clarify that “persistently abnormal lab parameters” was a **predictor** found to be *associated with* the unfavourable outcome (which was defined separately by WHO criteria), not that it was used to *define* the outcome itself.
6. Line 294-299: The limitation regarding data from medical charts is well-stated. The authors could further strengthen this by explicitly mentioning the potential for unmeasured confounding (e.g., socioeconomic factors, health literacy) which is a common limitation in retrospective studies based on routine data.

**Conclusion**

This is a strong manuscript that makes a significant contribution to the literature on TB outcomes in an understudied but important region. Addressing major comments as listed above will further enhance the impact of this paper. I am pleased to recommend it for publication after these minor revisions.
